# Ontogenetic characterization of sporangium and spore of *Huperzia serrata*: an anti-aging disease fern

**DOI:** 10.1186/s40529-016-0151-9

**Published:** 2016-11-14

**Authors:** Hua Long, Jing Li, You-You Li, De-Yu Xie, Qing-Zhong Peng, Li Li

**Affiliations:** 1 Key Laboratory of Plant Resources Conservation and Utilization, College of Biology and Environmental Sciences, Jishou University, Jishou, 416000 Hunan Province China; 2 Department of Plant and Microbial Biology, North Carolina State University, Raleigh, NC 27695 USA

**Keywords:** *Huperzia serrata*, Sporangium, Spore, Ontogenesis

## Abstract

**Background:**

*Huperzia serrata* is a medicinal plant used in Traditional Chinese Medicine, which has been used to prevent against aging diseases. It is mainly propagated by spores and grows extremely slowly. Due to severe harvest, it is a highly endangered species. In this report, we characterize ontogenesis of sporangia and spores that are associated with propagation. A wild population of *H. serrata* plants is localized in western Hunan province, China and protected by Chinese Government to study its development (e.g. sporangia and spores) and ecology. Both field and microscopic observations were conducted for a few of years.

**Results:**

The development of sporangia from their initiation to maturation took nearly 1 year. Microscopic observations showed that the sporangial walls were developed from epidermal cells via initiation, cell division, and maturation. The structure of the mature sporangial wall is composed of one layer of epidermis, two middle layers of cells, and one layer of tapetum. Therefore, the sporangium is the eusporangium type. Spore development is characterized into six stages, initiation from epidermal cell and formation of sporogenous cells, primary sporogenous cell, secondary sporogenous cell, spore mother cell, tetrad, and maturation.

**Conclusion:**

The sporangial development of *H. serrata* belongs to the eusporangium type. The development takes approximately 1 year period from the initiation to the maturation. These data are useful for improving propagation of this medicinal plant in the future.

## Background

*Huperzia serrata* in the Huperziaceae family is a medicinal plant used in Tradition Chinese Medicine (Ma et al. 2005). This plant produces Huperzine A and its analogous alkaloids, which are important natural products used to prevent against and to treat aging diseases in China (Ortega et al. 2004; Tan et al. 2003). However, the growth of this species is extremely slow. In addition, its vegetative propagation is very difficult. Due to massive harvesting for isolation of *Huperzia* alkaloids (Tan et al. 2000, 2002a, b), this species and its relative species in the genus are extremely endangered in China.

To date, knowledge regarding *H. serrata* development, reproduction, and ecology is limited. Anatomic studies remain to be investigated in detail. These data are significant to enhance growth and propagation of this medicinal plant and its relatives in the field. In present study, the development of sporangium and spore of *H. serrata* was studied using microscopic technology. The ontogenesis, shape, and structures of spores were characterized in detail. All observations are useful to not only enhance the understanding of sporangium of ferns, but also provide basic information for propagation of this medicinal fern in the future.

## Methods

*Huperzia serrata* is native to the west region (called Xiang Xi) of Hunan Province, China. It distributes in the regions with altitudes of 800–1500 m sea level. A Natural Reservation Station, namely Gao-Wang-Jian Forest located in Gu Zhang County (28°37′42.4″N; 110°00′28.4″E; At: 904 ± 5 m), which is next to Zhangjiajie, the International Heritage Park in the west of Hunan Province, has been defined by Chinese Government. A wild *H. serrata* population was localized in this area and protected by Chinese Government for preservation. In addition, this location has been developed into a research station for ecological, plant development, and propagation researches.

Plants start to develop sporangia in the late March every year in the field. To characterize sporangium and spore development, plants and sporangia were photographed and then samples were collected every 15 days. The sporangia from multiple individual plants were collected and immediately fixed with FAA agent (50% ethanol:glacial acetic acid:formaldehyde = 89:6:5). The fixed samples were stored in the room temperature till use.

Fixed sporangia materials were washed six times with double distilled water. The cleaned tissues were then immersed in the Ehrlich’s hematoxylin (Shanghai Chemical Reagent Co., Ltd.) solution for 1 week until complete staining of tissues. Stained tissues were then dehydrated using a series of gradient concentrations of ethanol from 20% through 30, 40, 50, 60, 70, 80, 90, and 95% to 100% for 30 min each. Dehydrated tissues were further treated with a series of concentrations of dimethylbenzene (20, 40, 60, 80, and 100%) for 30 min each. Finally, all treated materials were embedded in paraffin and cut into 5–8 μm thick sections by a microtome (MICROM HM310). Each section was placed on a clean slide, covered with a cover slip, and then mounted with Canada balsam. Each section was examined under Leica DM2000 microscope using objective lenses with different magnifications and images of cells were photographed.

## Results and discussion

### Formation stages and morphological changes of sporangium during development

**Fig. 1:**
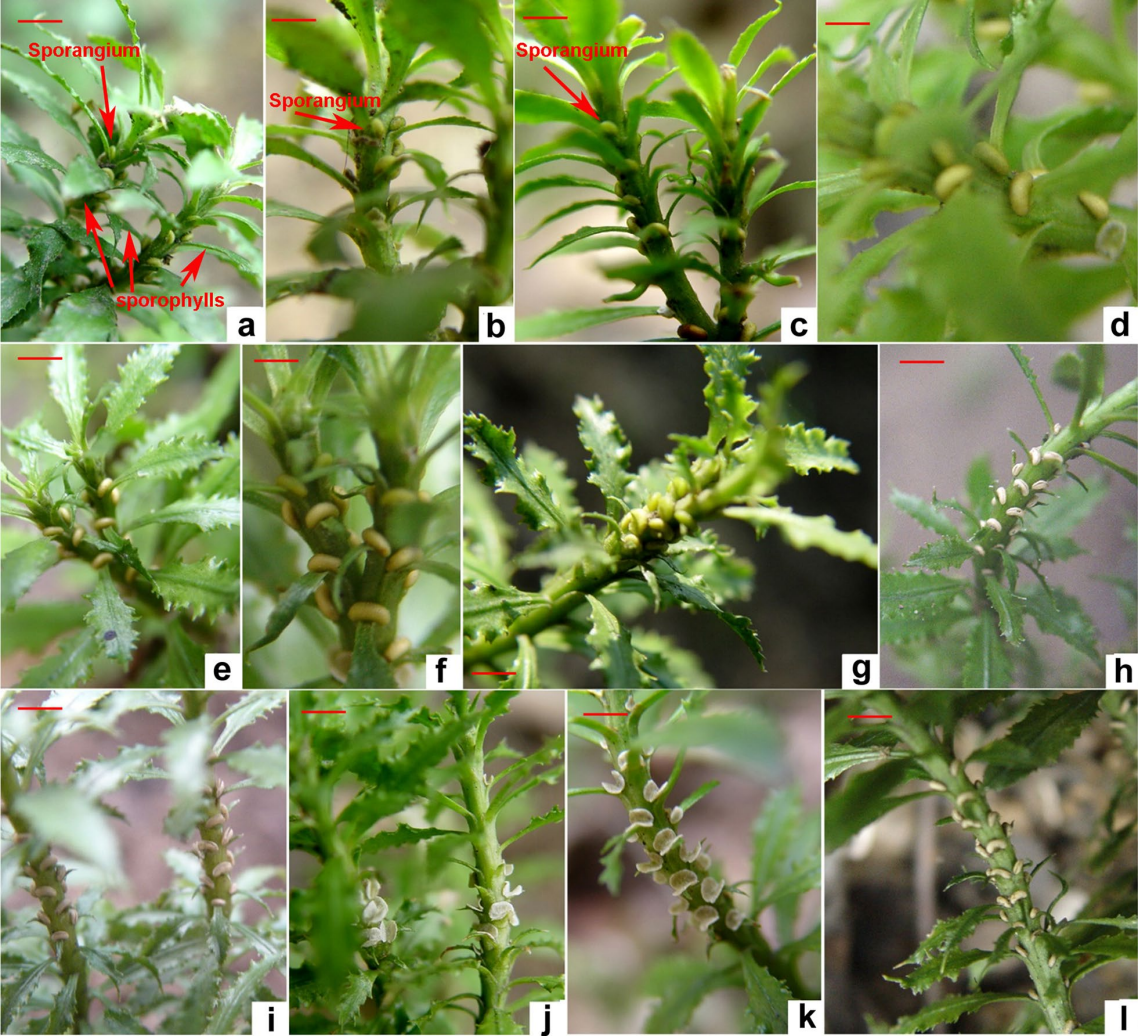
Morphologies of sporangia during plant development. **a** An early stage of kidney-shaped sporangia developed from the auxiliary side of leaves in the late April; **b**–**j** morphologies of growing sporangia in the middle May (**b**), the early June (**c**), the early July (**d**), the late August (**e**), the middle September (**f**), the middle October (**g**), the middle November (**h**), the middle December (**i**), the late January of the next year (**j**); **k**, **l** the residues of sporangia after spores detach. *Bar* 12 mm in **a**, **h**, **i**, and **l**; *bar* 8 mm in **b**, **c**, **e**, and **g**; *bar* 6 mm in **f**, **g**, and **k**; *bar* 5 mm in **d**

Zygotes started to germinate sporophytes in the late March or early April. When young sporophytes grew to 2–3 cm in height in the field, they started to dichotomously branch. These two new branches did not grow equally in size and length, one big and the other small, which is referred as dichotopodium branching (Bock 1962). The morphologies of sporophyll and sterile fronds (vegetative leaves) are undistinguished until the development of sporangia. According to our observation, leaves developed in the early March did not form sporangia; while, leaves developed from branches after the late March would form sporangia. The genesis of sporangia commonly occurred in the axil of sporaphylls (new fertile fronds or leaves) developed in early April and early May every year. Based on continuous observation for several years, we observed that most of leaves developed from early April through early May are sporophylls (or fronds) (Fig. 1a).

In the past several years, we observed that sporangia were developed at the axil of sporophylls, one sporangium per sporophyll. In general, the formation of sporangia initiated in late March. Sporangia were hardly seen by naked eye during the initiation, until the late April when a small kidney-shaped and green structure appeared from the axil of leaves (Fig. 1a). From the late April to the middle May, sporangia grew to an obvious structure (Fig. 1b), although the growth rate was slow. From the late May to the middle June, the size of sporangia rapidly increased and the surface color became deeper greenish (Fig. 1c). This growth did not stop until the early July when sporangia developed into kidney-shaped structure and the surface pigmentation started to change from green to light yellow (Fig. 1d). Then, sporangia stopped growth in size. The yellowish coloration of sporangia became deeper in August (Fig. 1e) through September (Fig. 1f). In September, sporaphylls continued to ripen. By the middle October, sporangia were fully mature (Fig. 1g), while fully ripe sporangia longitudinally opened to spread spores and then detached from stems. These activities continued in November (Fig. 1h), and December (Fig. 1i) through the next January (Fig. 1j). Occasionally, some plants did not completely spread all spores until the early March of next year. After spores were completely released, the left walls of many sporangia detached from the stem from January through March of the next year (Fig. 1j–l). Therefore, it takes almost 1 year from the initiation to completeness of spore dispensing.

### Sporangial primordia observed by microscopic observation

**Fig. 2:**
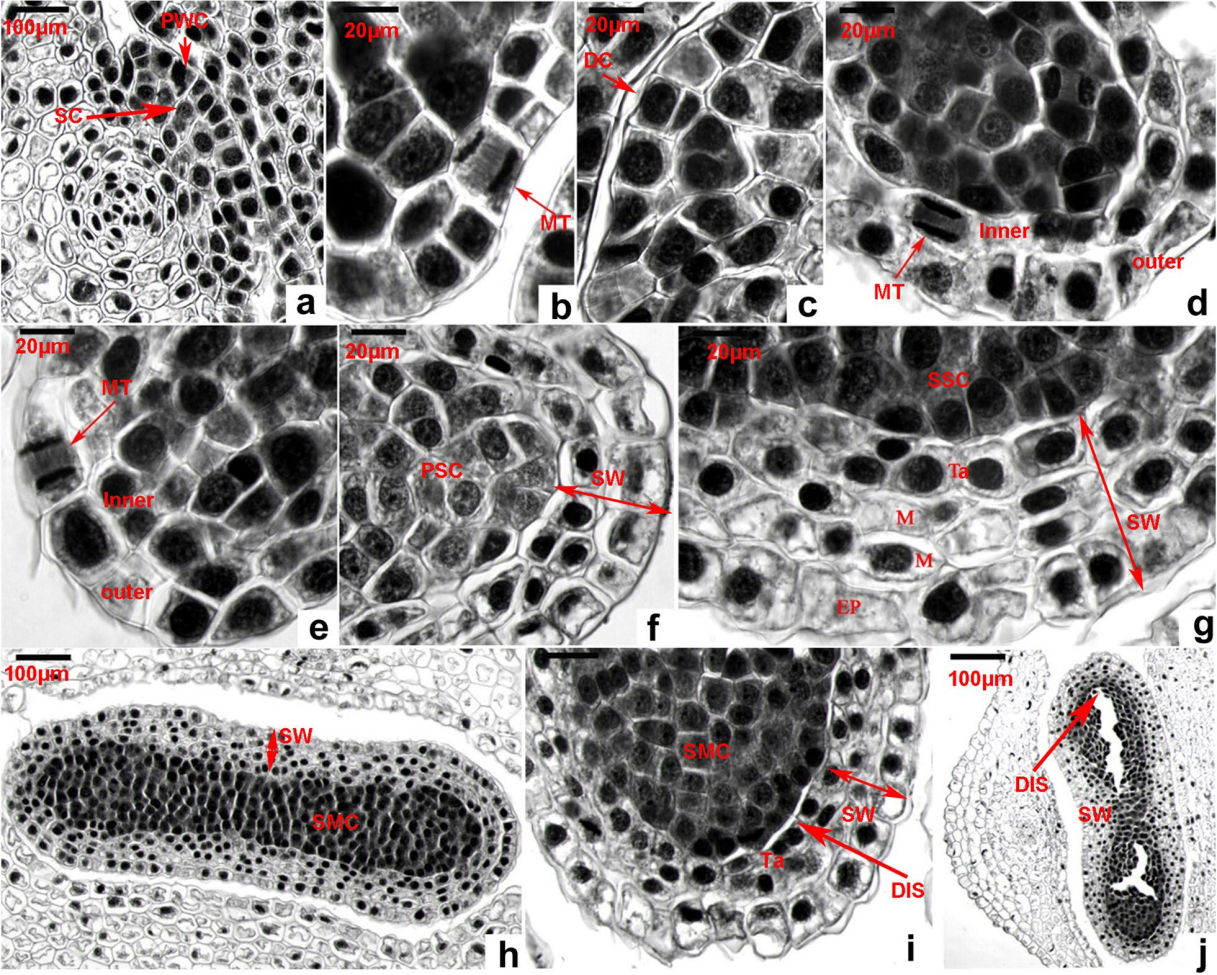
The formation and differentiation of sporangium wall. **a** A sporangial primordium includes primary wall cells (PWC) and sporogenous cell (SC) stained with deep density. **b** An epidermal initial cell is performing a periclinal division indicated by an *arrow*. *MT* mitosis. **c** An epidermal cell is divided into two daughter cells (DC), one inner daughter cell to forming sporogenous cell and one outer daughter cell forming primary mantle cells in the late development. **d** An image shows a periclinal division (indicated by an *arrow*, *MT* mitosis) of a cell derived from the primary mantle cells, the result of which increases the number of layer. Those deep stained cells next to this dividing cells develop into spores. **e** An epidermal cell derived from the mantle cells is performing an anticlinal division (indicated by an *arrow*, *MT* mitosis) to expand the number of the outmost cell layer. **f** This image shows a 2–3-cell layer (indicated by a *two-end arrow*) forming the wall structure (*SW* sporangium wall) of a sporangium during the primary sporogenous cell (PSC) stage. **g** This image shows a 4-cell layer (indicated by a *two-end arrow*, *SW* sporangium wall) mature wall of a sporangium during the stage of secondary sporogenous cells (SSC); *EP* epidermis, *M* middle layer, *Ta* tapetum. **h** An image of a longitudinal section of one sporangium shows structures of a mature wall (*SW* sporangium wall) and spore mother cells (SMC) during the stage of secondary sporogenous development. **i**, **j** Images show disruption (indicated by DIS) of the sporangial wall in the stage of spore mother cells (SMC)

We collected sporangia samples from the early March to the next January for light microscopic observation. Microscopic observation on a series of sections revealed that each sporangium starts with two layers of cells, which are characterized by high density of staining and similar cell shape from the axil of sporophylls (Fig. 2a). This group of cells form a sporangial primordium that includes an outer layer and an inner layer of cells. As described below, the inner layer (sporogenous cells) is associated with the formation of sporogenous cells and tapetum, the late of which is part of sporangial wall. The outer layer (primary wall cells, PWC) is associated with the formation of the sporangial wall described below.

### Observation of cell division from primary wall cells and formation of sporangial wall

Mitosis (Fig. 2b) in the periclinal direction from the PWC formed two daughter cells, one inner and the other outer (Fig. 2c). Successive observation indicated that the inner one is associated with the formation of the inner layer of cells, while the outer one is associated with the formation of the outer layer of cells (Fig. 2d). Continuous periclinal cell division from the inner layer (Fig. 2d) was observed to result in more layers of cells (Fig. 2f). In addition, continuous anticlinal cell division from the outer layer (Fig. 2e) was observed to result in expansion of the epidermis. We have observed a large number of successive dissection slides and found that the inner layer cells only performed 1–2 periclinal division. Continuous cell divisions resulted in 2–3 layers of cells to form a sporangial wall structure (Fig. 2f).

**Fig. 3:**
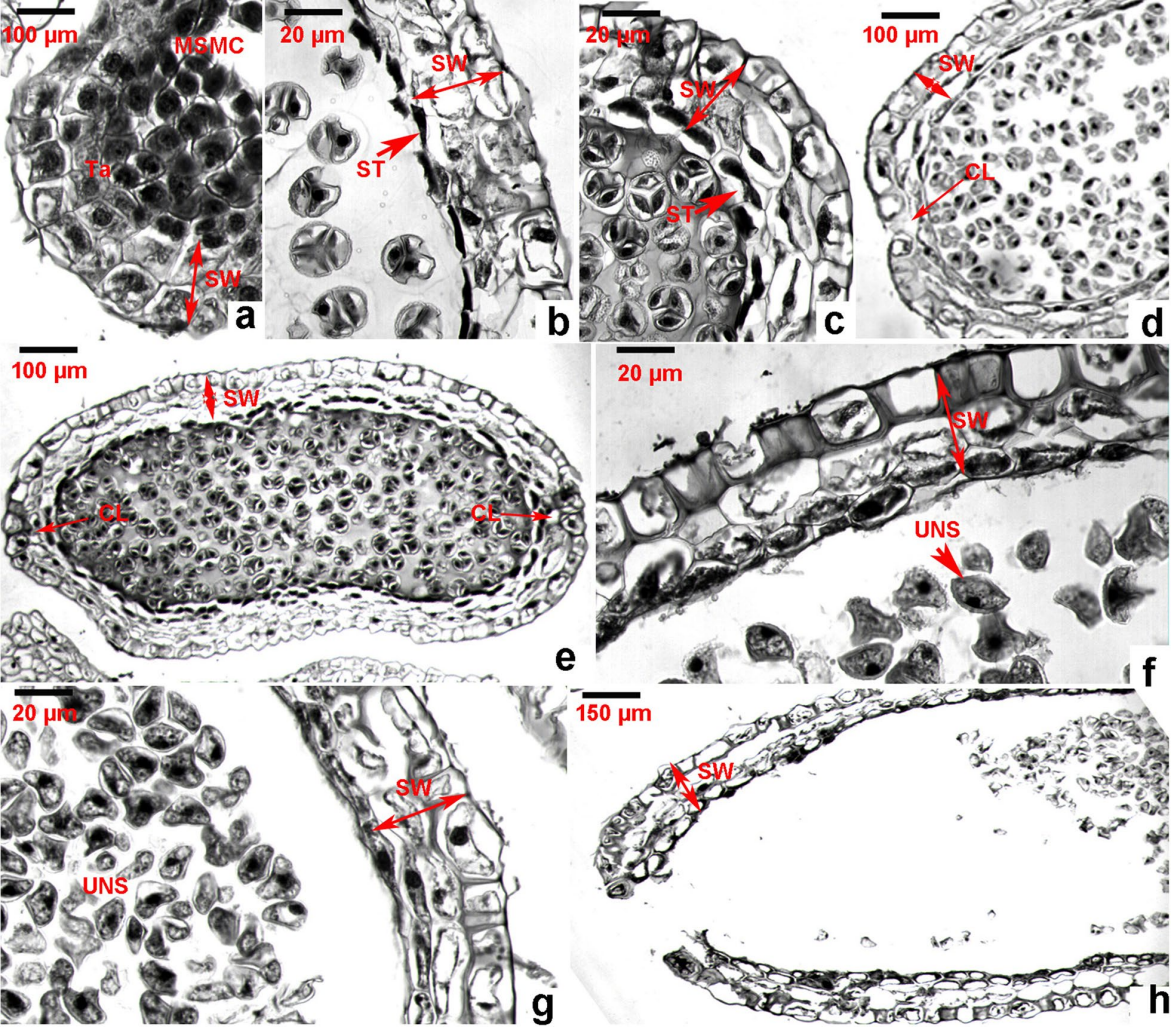
Morphological changes of sporangium wall from the spore formation through release. **a** At the stage of mature spore mother cell (MSMC), showing initiation of cell degeneration from tapetum (Ta); **b**, **c** disruption of secretory tapetum (ST) in the sporangium wall (SW); **d**, **e** the constricting location (CL) from the sporangium wall (SW) of kidney-shaped sporangia; **f**, **g** the feature of the sporangium wall (SW) in the stage of uninucleate spores (UNS); **h** disruption of the sporangium wall (SW)

Tapetum has been reported to originate from the inner layer cells of the sporangial primordia (Davis 1966; Foster and Gifford 1959; Foster and Gifford 1974). Based on our microscopic observation, the layer of tapetum (Fig. 2g) is associated with the inner sporogenous cells of the primordia (Fig. 2a). Their morphology is different from those cells resulted from the middle and epidermal cells (Fig. 2g). From the top overview of dissections, the tapetum cells are rectangular and stained intensively (Fig. 2g). The tapetum cells are associated with spore development described below. The disruption of tapetum was observed during the early stage of the spore formation, such as the early spore mother cell development stage (Figs. 2i, j, 3a). One of tapetum functions is to store nutrients and then secret them during spore development (Hesse et al. 1994; Oldenhof and Willemse 1999; Willemse and Reznickova 1979). This observation indicates that the tapetum cells secret contents to spore mother cells. Based on the classification for sporangium development types of lower vascular plants defined by Goebel (Goebel 1905), the tapetum of *H. serrata* belongs to the secreting tape.

By the time of nearly mature of sporangia, the sporangial wall consists of 4–5 layers of cells, including the outermost epidermis, two middle layers, and the innermost layer of tapetum (described below) (Fig. 2g). From the top overview, the shapes of epidermal and middle layer cells are either square or rectangular. The shape of most of the tapetum cells is rectangular (Fig. 2g). By this stage of development, sporangia are kidney-shaped from the longitudinal view (Figs. 1a, 2h). Based on categorization theory proposed and defined by Bower (1891, 1896) and supported by Fagerlind (1961), the development of sporangial wall belongs to the eusporangiate type. Our observation also support the sporangial wall development reported for *Huperzia brevifolia* (Baron et al. 2009).

The structural changes of sporangial wall were continuously observed after the formation of sporangia in March (Fig. 1a–g). Microscopic observation from numerous slides showed that since May (Fig. 1b), the degeneration of the two layers of middle cells was observed (Fig. 3b, c). This change was observed along with the starting of disruption of the tapetum associated with the formation of tetra structures. In addition, microscopic observation on successive slides showed that two middle layers of cells completely degenerated or only remained a trace residue (Fig. 3f, g).

The structural changes of epidermis of the sporangial walls were also observed. During maturation of sporangia, a layer of cuticle was observed on the surface. The contents of epidermal cells gradually decreased during the maturation, which was clearly shown by lighter density of cell staining (Fig. 3f–h). After full maturation, openings in the longitudinal direction of sporangia were observed (Fig. 3h), from which spores were released to the environment.

### Observation of cell division from sporogenous cells and formation of spores

**Fig. 4:**
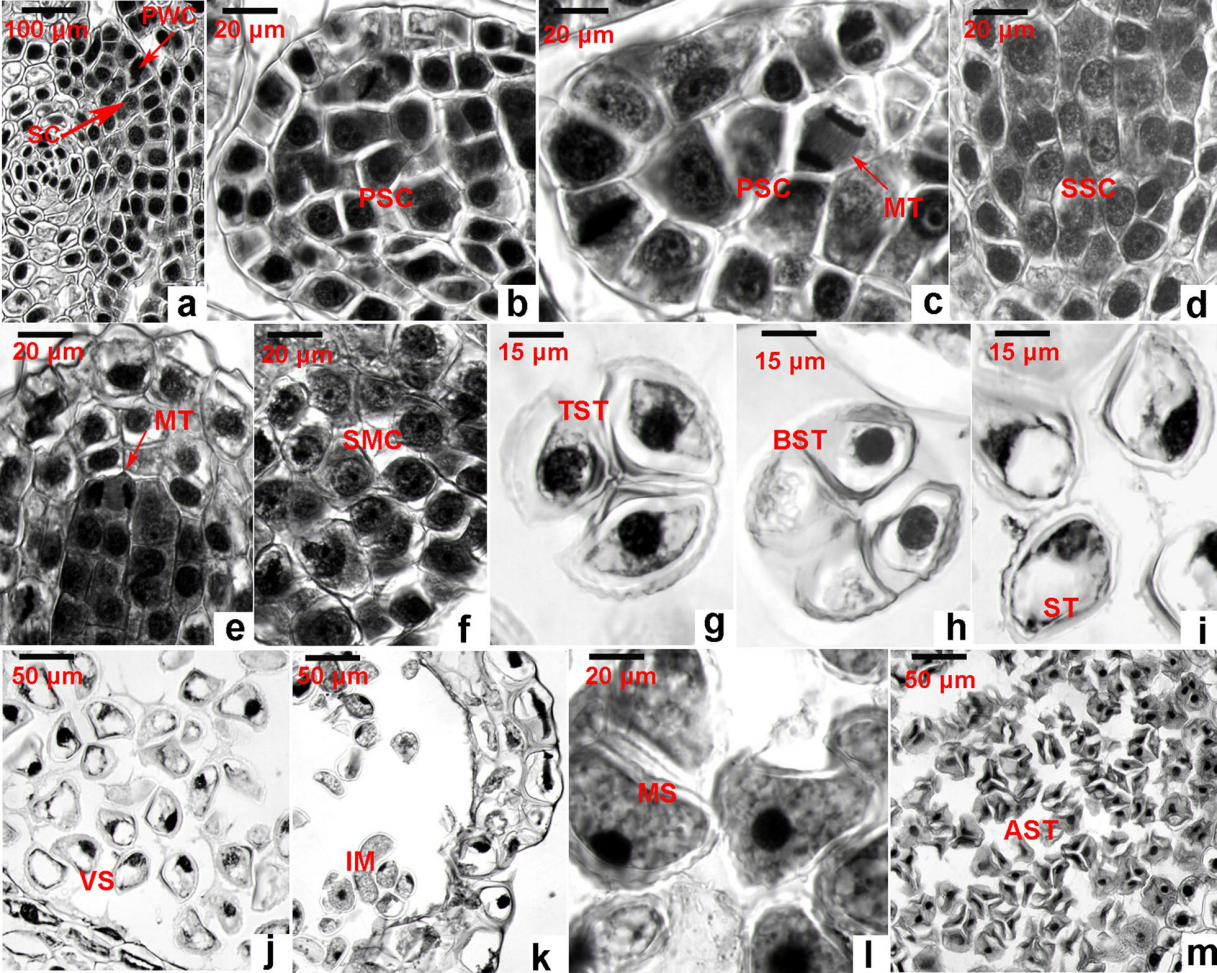
Development of spores. **a** Primary wall cells (PAC) and sporogenous cells (SC); **b** primary sporogenous cells (PSC); **c** mitosis (MT) of primary sporogenous cell; **d** secondary sporogenous cells (SSC); **e** mitosis (MT) of a secondary sporogenous cell; **f** spore mother cells (SMC); **g** tetrahedral spore tetrads (TST); **h** bilateral spore tetrads (BST); **i** spore tetrads (ST); **j** vacuolated spores (VS); **k** immature spores (IM); **l** mature spores (MS); **m** abortive spore tetrads (AST)

Our microscopic observation revealed six types of structural changes from the sporogenous cells to mature spores. Based on the occurring order, we characterized these processes into six stages, sporogenous cell (initiation and formation), primary sporogenous cell, secondary sporogenous cell, spore mother cell, tetrahedroid (tetrad), and mature spore stages (Fig. 4). As described above, sporogenous cells are the inner layer cells derived from the epidermal cells of axil of sporophylls (Figs. 2a, 4a). Periclinal division of cells were observed in our microscopic observation of successive slides. In addition, anticlinal cell division was observed to increase cell numbers (Fig. 4c). The resulting daughter cells were morphologically similar, named as primary sporogenous cells (Fig. 4b, c). The layer of cells next to primary wall cells developed into tapetum as described above. The remained multiple cells, namely primary sporogenous cells that were featured by high density with staining (Fig. 4b, c), continued cell division to increase cell numbers, which were named as secondary sporogenous cells featured by high density stained (Fig. 4d). Continuous mitosis (Fig. 4e) increased new daughter cells, which were featured by light cell density stained and a clear vacuole observed under microscope (Fig. 4f), thus were named as spore mother cells in this study. Based on our successive observation, the stage of spore mother cell formation took a relatively long time. Unfortunately, we could not obtain a mitosis image during spore mother cell formation. After the stage of spore mother cells, we observed two tetrad structures in the sporangial chambers from successive dissection slides. They were tetrahedral spore tetrads (Fig. 4g) and bilateral spore tetrads (Fig. 4h). In addition, other shapes were observed (Fig. 4i). A typical mature tetrad includes four spores (Fig. 4g). Based on other reports, tetrads are covered by callose (Albert et al. 2011). Followed by these morphologies, the disruption of tetrads was observed in immediate successive dissection slides. This resulted in release of spores into the sporangial chambers. Spores are characterized by a clear vacuole, cytoplasm, and a nucleus (Fig. 4j). From our observation, these spores, called immature spores, were not released until full maturation and the opening of sporangial wall (Fig. 4k, l). After release of spores from fully ripe sporangia, the remaining abortive spore tetrad structures were still observed from dissection slides (Fig. 4m).

This medicinal plant is not easy to be propagated to obtain a large biomass for Huperzia alkaloids. The main approach of growth is through spores. The results shown here provide important information that helps improve propagation of this plant in the future. We expect to develop technologies to shorten the time for sporangium development.

## Conclusion

The sporangial development takes approximately 1 year. After a few years of observation and microscopic analysis, we characterize that the sporangia of *H. serrata* belongs to the eusporangium type.

## Abbreviations

PWC: primary wall cell
SC: sporogenous cell
DC: daughter cells
PSC: primary sporogenous cell
SW: sporangium wall
